# Infestation and Larval Habitat Ecology of *Aedes aegypti* and *Aedes albopictus* in an Urban Gradient in Vassouras, Rio de Janeiro, Brazil

**DOI:** 10.3390/insects16080869

**Published:** 2025-08-21

**Authors:** Gilliarde de Carvalho Caetano, Samanta Cristina das Chagas Xavier, Mariana Rocha David

**Affiliations:** 1 Laboratório de Mosquitos Transmissores de Hematozoários, Instituto Oswaldo Cruz, FIOCRUZ, Rio de Janeiro 21040-360, Brazil; gilliardcarvalhos@hotmail.com; 2 Laboratório de Biologia de Tripanosomatídeos, Instituto Oswaldo Cruz, FIOCRUZ, Rio de Janeiro 21040-360, Brazil; samanta@ioc.fiocruz.br

**Keywords:** *Aedes*, entomological surveillance, vector control, mosquito ecology

## Abstract

Since there are no available vaccines against some arboviruses, vector control is the most effective way to reduce their incidence. In this context, mechanical control is one of the most cost-effective ways to suppress *Aedes* populations, but it requires knowledge about vector breeding ecology in varied landscapes and climates. Here we investigated the infestation levels and container types used as larval habitats by *Aedes aegypti* and *Aedes albopictus* in an urban gradient of Vassouras, a countryside city in Rio de Janeiro. Larval surveys were conducted bimonthly from January 2017 to December 2018. Infestation was measured through the House (HI) and Breteau indexes (BI). Container types found with *Aedes* spp. immatures were correlated with temperature and rainfall and were compared between urbanization zones. The distribution of positive containers was mapped. The HI for *Ae. aegypti* increased during rainy seasons, but the HI and BI were always <1% for both mosquito vectors. More reservoirs were found harboring *Ae. albopictus* than *Ae. aegypti*, but in general their relative distribution into types was similar between species. On the other hand, the amount and distribution of containers into types varied across urbanization zones. Finally, the spatial distribution of larval habitats was similar between species, as well as often constant between seasons and study years.

## Simple Summary

*Aedes aegypti* and *Aedes albopictus* are mosquitoes that transmit viruses such as dengue, Zika and chikungunya. Since there are no vaccines for some of these diseases, mosquito control is the best strategy to reduce their spread. This study analyzed where the larvae of these mosquitoes develop in different urban areas of Vassouras, a countryside city in Rio de Janeiro. Between 2017 and 2018, we monitored the presence of larvae in different types of containers and measured the infestation levels across seasons. The results showed that infestation was low, with a significant association between the abundance of *Ae. aegypti* larval habitats and rainfall. *Aedes albopictus* was found more frequently than *Ae. aegypti*, but both species used similar types of water reservoirs as larval habitats. The quantity and type of containers varied depending on the degree of urbanization. The spatial distribution of larval habitats remained relatively stable over time. This information is useful for guiding mosquito control efforts, helping to reduce the risk of arbovirus outbreaks.

## 1. Introduction

The mosquitoes *Aedes aegypti* and *Aedes albopictus* cause concern among health authorities because they transmit several arboviruses to human populations, such as dengue (DENV), chikungunya (CHIKV) and Zika (ZIKV), which is facilitated by their anthropophilic behavior [1]. Around 390 million people are infected with DENV annually worldwide [2] and Brazil is historically the country with the highest number of cases [3,4,5]. Recently, CHIKV and ZIKV arrived in the country and caused massive epidemics [6,7]. Both vector species are exotic in South America: *Ae. aegypti* arrived during the 15th century on ships bringing enslaved Africans [8], while *Ae. albopictus* was later introduced to the continent in the 1980s, possibly through ships from Asia that arrived in the Espírito Santo state [9], being formally detected in Brazil in 1986 [10].

Since there are no widely available vaccines against some arboviruses that infect humans, such as ZIKV and CHIKV, vector control is still the most effective way to reduce the incidence of arboviral infections. Its main objective is to reduce vector populations below a threshold sufficient to sustain epidemics [11]. In Latin America, *Ae. aegypti* is the primary vector of DENV, ZIKV and CHIKV in anthropic environments and *Ae. albopictus* is considered a secondary vector, as it is susceptible to those viruses under laboratory conditions but adult females are sporadically found naturally infected [12]. Both species are well adapted to the urban environment. *Aedes aegypti* breeds mainly in man-made containers inside and around houses [13] and prefers to feed on human blood [14,15]. On the other hand, *Ae. albopictus* has been associated with vegetated areas, where it lays eggs in natural and artificial water reservoirs around houses and on forest edges, parks and botanical gardens [16]. It blood-feeds on humans and also on other mammals such as dogs and rats. Due to this behavior, *Ae. albopictus* is called a bridge vector, as it can carry pathogens between sylvatic and anthropic environments [17].

Vector control can be achieved in different ways, such as through chemical (i.e., via insecticides), biological (i.e., using pathogens or predators) or genetic control (e.g., transgenesis and the release of insects carrying dominant lethal genes) [18]. Arbovirus transmission reduction has also been achieved through the use of bacterial symbionts, such as *Wolbachia* [19], but one of the most cost-effective ways to reduce *Aedes* populations in urbanized areas is mechanical control, which is based on eliminating or preventing the access of female mosquitoes to artificial containers used by those species as larval habitats [20]. However, this methodology faces some challenges, such as the lack of appropriate infrastructure in big cities to guarantee regular water supply and garbage collection, which results in a great availability of larval habitats such as water barrels for domestic use and discarded plastic materials [21]. Moreover, citizens must be engaged in the continual elimination of *Aedes* larval habitats in their own houses, since the inspection of all premises for mechanical control by government health agents is unfeasible [22].

In this context, optimized mechanical control must rely on massive population engagement encouraged by locally specific educational campaigns. Control efforts and community engagement should focus mainly on most-used containers by mosquitoes, i.e., those water reservoirs frequently found holding *Aedes* immatures, which are often determined by seasonality, human behavior and urban infrastructure [23]. In Brazil, many studies on the breeding ecology of *Ae. aegypti* and *Ae. albopictus* investigating their occurrence in natural/artificial containers inside and around houses are carried out in highly urbanized metropolitan regions, such as Rio de Janeiro, São Paulo and other state capitals, where most arboviral cases are registered [23,24,25,26]. In contrast, we present data from a city in the interior of the state of Rio de Janeiro, with a mostly rural territory.

## 2. Materials and Methods

### 2.1. Study Site

**Figure 1:**
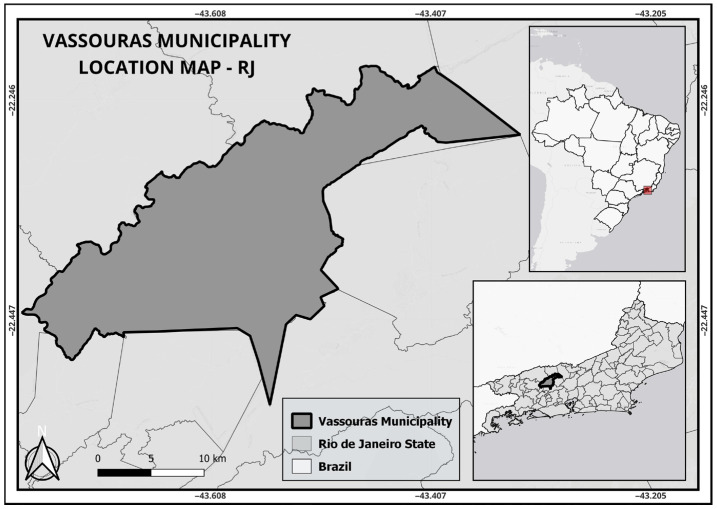
Geographical location of the study area in Vassouras Municipality, Rio de Janeiro, Brazil. (base map modified from IBGE available from [28]). The red box delineates the geographic location of the municipality of Vassouras, in the state of Rio de Janeiro.

The study was conducted in 2017 and 2018 in the municipality of Vassouras (−22.404291° S; −43.657879° W), State of Rio de Janeiro, Brazil (Figure 1). This city has an area of 552.4 km^2^, 19,574 premises (City Hall and the Municipal Health Secretariat (MHS) of Vassouras), 33,976 inhabitants [27] and a population density of 63.3 inhabitants/km^2^. It is located on the shore of the Paraíba do Sul River, at an altitude of 434 m. The region has a high-altitude tropical climate (Cwa), with an average monthly temperature ranging from 16 °C to 28 °C and an average monthly rainfall of 80.7 mm.

**Figure 2:**
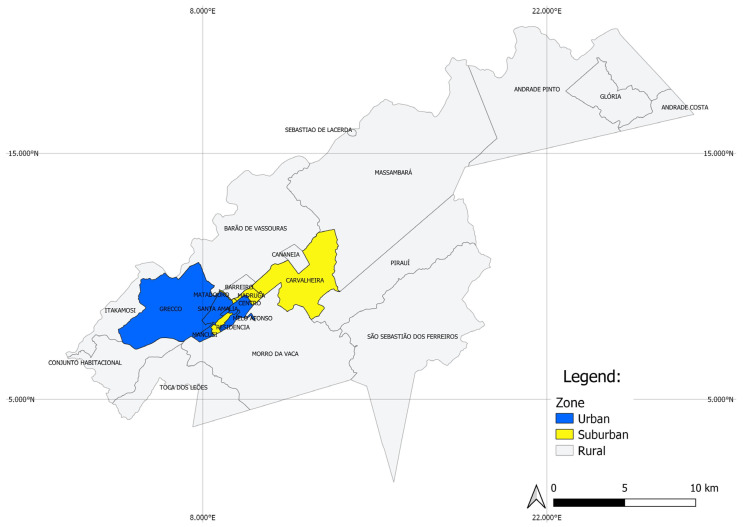
Vassouras map with delimitation in urban, suburban and rural zones.

Vassouras is located in Vale do Café (or Coffee Valley), a region of importance for local tourism due to historical coffee farms. It has a small urban center surrounded by an extensive rural territory. The city is divided into 24 neighborhoods, which were classified according to the degree of urbanization into urban, suburban and rural (Table 1, Figure 2). Urban neighborhoods comprised 11,399 premises (including houses, buildings, hospitals, places of worship and commercial establishments) and were characterized by the presence of high-standard houses, with two to three bedrooms and decorated backyards, as well as reliable water supply and garbage collection. Suburban sites comprised 2728 premises. This is the most populous area, with high concentration of residences and commerce, irregular construction and precarious infrastructure and basic sanitation. Despite having paved streets, there are also some common characteristics of rural areas, such as the proximity of houses to forest fragments, larger backyards compared to those in the urban area and animal husbandry, such as chickens and other birds. The rural area has the greatest territorial extent (~90% of the city territory), holding 5447 premises. This land is mostly used as grazing land for cattle. There are also small farms for animal husbandry, where animals are usually maintained close to the residences, which generally have a good structure, but not always adequate basic sanitation (regular water supply, sewerage and garbage collection).

During field collections, 104 and 6 probable human infections (i.e., diagnosed based on clinical symptoms) by DENV and CHIKV were registered, respectively. Epizootics of yellow fever were recorded in non-human primates from sylvatic areas (data from the Rio de Janeiro State Health Department).

### 2.2. Climatic Data

Daily temperature and precipitation data for the municipality of Vassouras from January 2017 to December 2018 were sourced from the Agrometeorological Monitoring System, Agritempo (https://www.agritempo.gov.br, accessed on 31 August 2022), using records from the Vassouras weather station (TRMM.1110), which operates under the World Meteorological Organization’s standards. For analytical purposes, daily records were aggregated into monthly averages for temperature (°C) and monthly totals for rainfall (mm). These summarized data were then used to characterize seasonal patterns and to identify periods of increased rainfall and temperature, which are known to influence *Aedes* mosquito proliferation. The climatological period was divided into a rainy season (November to April) and a dry season (May to October) for spatial analysis, in accordance with the region’s Cwa climate classification (high-altitude tropical climate with dry winters).

### 2.3. Entomological Survey

Larval surveys were conducted bimonthly inside all premises and in the peridomestic area during vector control activities routinely carried out by health agents from the Municipal Health Secretariat (MHS) of Vassouras from January 2017 to December 2018. Upon finding a water reservoir with mosquito larvae, the health agent collected five specimens with the aid of a plastic dropper-type pipette (Pasteur), placing them in a plastic tube with 70% alcohol. The tubes received an identification tag and were sent to the MSH lab for taxonomic identification of larvae. The collection date, address and the type of container where the larvae were found were registered in a field form. Water deposits were classified into five types (A–E) according to the nomenclature established by the Brazil Ministry of Health [29]. Type A comprises large water storage reservoirs holding water for domestic use (such as water tanks, cisterns and ground reservoirs such as barrels). Type B includes mobile reservoirs that are useful/important for residents and cannot be eliminated from the environment, such as vases, reusable bottles, thaw containers from refrigerators, animal water pots and religious objects. Type C includes fixed reservoirs, such as gutters, disused toilets, non-treated pools and flower vases in cemeteries. Type D refers to reservoirs that can be discarded, such as tires, plastic bottles, disposable cups, food packaging and cans. Finally, type E includes natural reservoirs, such as bromeliads, tree holes and rocks. Larval habitats were eliminated, covered or, when this was not possible, treated with larvicide.

After the tubes arrived at the laboratory, the collected pupae and larvae were identified under an optical microscope (XSZ107BN, Coleman, Santo André, Brazil) in 10× objective to identify the mosquito species. Immatures were removed from the tube with the aid of a brush and placed first in a Petri dish (10 cm in diameter and 1 cm in height) to assess the larval or pupal stage and anatomical conditions. Specimens that had their anatomical structures damaged were discarded. After these steps, insects were transferred to a glass slide (7 cm long and 2 cm wide) with the aid of a brush and observed under a magnifying glass for taxonomic identification according to the taxonomic keys proposed by [9]. The results were registered in an Excel data sheet. Positive containers were identified by collection data and address and registered in separate spreadsheets for *Ae. aegypti* and *Ae. albopictus*, so it was not possible to identify water reservoirs that contained both species simultaneously. Other mosquito species were classified as “other” following the *Aedes* surveillance protocol established by the Brazil Ministry of Health [29].

### 2.4. Data Analysis

Mosquito infestation was evaluated through the House Index (HI) and Breteau Index (BI) [11]. The HI expresses the percentage of positive premises for immature forms of *Ae. aegypti* and *Ae. albopictus* and was calculated as the number of infested premises/inspected premises × 100. According to the National Guidelines for the Prevention and Control of Dengue Epidemics (2009) [30], municipalities can be classified according to the HI of *Ae. aegypti*, with municipalities with an HI of 0 to 0.9% being considered satisfactory, those with an HI of 1.0% to 3.9% on alert and those cities with an index above 4.0% as being at risk of occurrence of arbovirus epidemics [29].

Although the HI is the main infestation evaluation index of *Ae. aegypti* in the country, this index does not take into account the number of larval habitats found in each premise. Thus, the Breteau Index (BI) was calculated as the number of positive water deposits/inspected premises × 100. It defines the average number of larval habitats found for each species per 100 houses inspected [11]. The HI and BI were calculated for the municipality of Vassouras for each cycle of the larval survey. Although these indices represent different entomological metrics, they showed highly similar values in our dataset due to the fact that, in most positive houses, only a single larval habitat was detected. Given this strong correlation, we opted to use only the House Index (HI) in the following modeling.

The association between the HI, climatic variables and city zones were tested separately for *Ae. aegypti* and *Ae. albopictus* using beta regression models, which are suitable for proportional data with heteroscedasticity and bounded between 0 and 1. Because the beta distribution is defined on the open interval (0, 1), values of 0 were replaced by 0.001 and values of 1 were replaced by 0.999 [31]. Since larval surveys were conducted bimonthly, we used bimonthly averages of temperature and total rainfall as explanatory variables, ensuring that the climatic data matched the temporal resolution of the entomological surveys. To account for potential interactions between city zones and climatic factors, interaction terms between zone and average temperature, as well as zone and average precipitation, were included in the candidate models. Multiple models with different combinations of main effects and interactions were tested. The best-fitting model for each species was selected based on the Akaike Information Criterion (AIC), choosing the model with the lowest AIC value as the most parsimonious. As larval habitats were eliminated or covered at each survey cycle, which minimizes the likelihood of repeated measures on the same breeding sites (i.e., each survey reflects a new sampling context) we initially assumed temporal independence between observations. However, to further support this assumption, we compared models with zone as a random intercept (i.e., that accounts for intra-zone correlation) versus as a fixed effect. If the random-effects model showed a substantially better fit (ΔAIC > 2), we concluded that observations could not be independent within zones and proceeded with model selection using a hierarchical structure. Model fit was examined by checking variance inflation factors (VIF), heteroscedasticity, residuals dispersion and the presence of outliers with the DHARMa R package (version 0.2.7) [32]. To assess the statistical significance of explanatory variables in the beta regressions, we performed Analysis of Variance (ANOVA). Type II tests were applied for models without significant interactions, while Type III tests were used for models including interaction terms. Predicted value plots were generated for variables and interactions with statistically significant effects on the HI.

Pairwise comparisons of the HI between city zones were performed for each species using Estimated Marginal Means (EMMs) derived from the selected beta regression models, with adjustments for multiple comparisons [33]. Additionally, the HI was compared within each city zone between *Ae. aegypti* and *Ae. albopictus* using a bootstrap resampling approach. Paired differences between species were resampled 10,000 times, and confidence intervals (CI) were computed from the bootstrap distribution to assess statistical significance.

To investigate the association between the number of each type of larval habitat (A to D) and environmental factors, we used Generalized Linear (Mixed) Models (GLM/GLMMs) with a negative binomial distribution to account for overdispersion. The response variable was the count of larval habitats of each type, while the explanatory variables included zone, bimonthly mean temperature and rainfall. Since observations for the two species were recorded in the same spatial unit (zone) overtime, we analyzed species separately to account for the lack of independence among observations. Multiple models with zone as a fixed or random effect and with different combinations of variables were tested, as explained before. Model selection was based on the AIC and model diagnosis was carried out using the DHARMa R package [32]. To assess the statistical significance of fixed effects, we performed Analysis of Variance (ANOVA). Finally, with the aim of assessing whether the proportion of container types differed between zones, the relative distribution of containers in types was compared for each species between zones using a chi-squared test. Data were transformed into x + 1 to avoid zeros in calculating the chi-squared statistic [34]. All analyses were performed in an R environment (version 3.6.2).

### 2.5. Geospatial Analysis

*Aedes aegypti* and *Ae. albopictus* larval habitats were identified and georeferenced using premise addresses. The geographic coordinates of each address were obtained using the Google Earth tool Version 7.1.4.1529, with the geodetic reference system WGS 84 (World Geodetic System 1984). Incomplete records, such as those lacking reservoir classification or species name, were discarded. Subsequently, the coordinates of every identified larval habitat location for either *Ae. aegypti* or *Ae. albopictus* were exported in shapefile format. To assess spatial patterns in larval habitat density across both rainy and dry seasons, Kernel density estimation was applied to generate heat maps, providing a spatial visualization of potential hotspots [35]. This approach complements the beta regression analysis by highlighting localized areas of elevated risk, thereby supporting targeted vector control at the neighborhood scale. All maps were produced and geospatial analysis was carried out using Quantum Software QGIS 3.10, a free and open source geographic information system software [35,36], using cartographic bases obtained from the Brazilian Institute of Geography and Statistics-IBGE (https://ibge.gov.br/ accessed on 3 March 2020).

### 2.6. Ethics

Larval surveys were performed by the health agents of Vassouras, whom householders were used to receiving in their home six times yearly during the surveillance routine following the guidelines of the Brazilian Dengue Control Program (PNCD) [37].

## 3. Results

### 3.1. Climate Data

**Figure 3:**
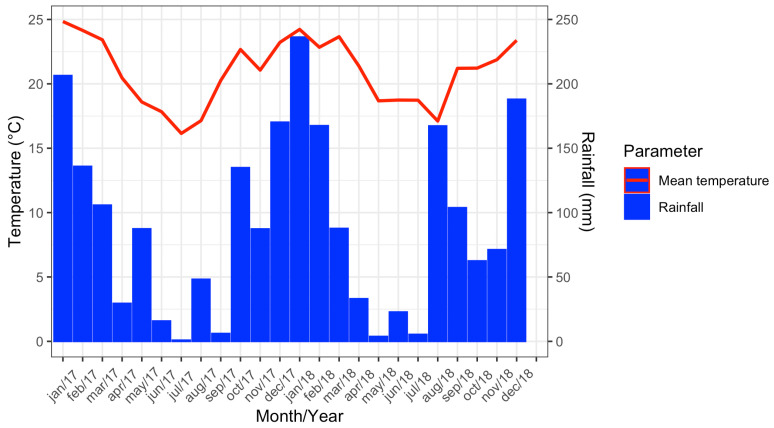
Climograph showing data from the meteorological station of Vassouras, Rio de Janeiro from January 2017 to December 2018.

In 2017, the maximum mean temperature recorded was 24.8 °C (January) and the minimum was 16.1 °C (July). The highest accumulated rainfall was in January, with 206.4 mm, while the lowest was registered in July (0.9 mm). For 2018, the maximum mean temperature recorded was 24.2 °C (January) and the minimum was 17.1 °C (August). In January it rained 236.3 mm, while the lowest accumulated rainfall was registered in May (3.8 mm) (Figure 3).

### 3.2. Aedes aegypti and Ae. albopictus Infestation Indexes

**Figure 4:**
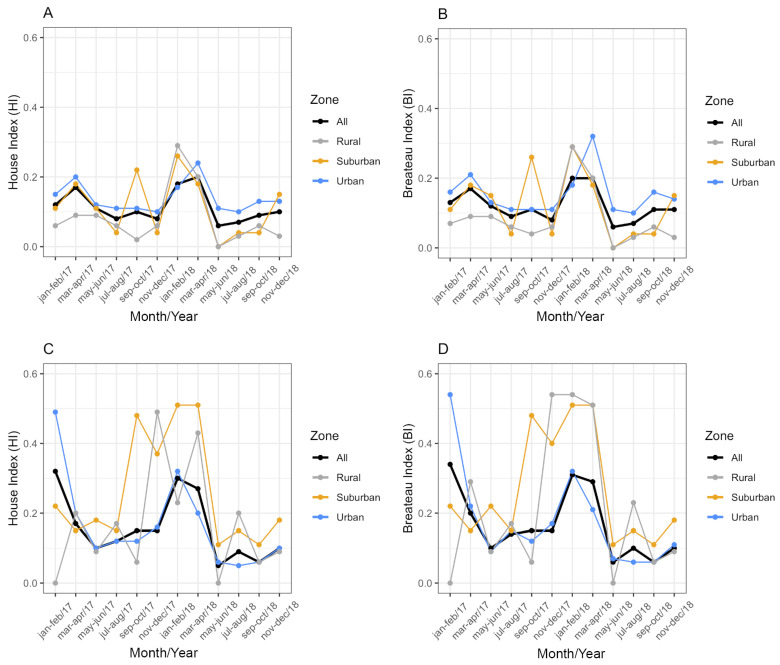
House index (HI) and Breteau index (BI) for *Aedes aegypti* (**A**,**B**) and *Aedes albopictus* (**C**,**D**) per urbanization zone in Vassouras from January 2017 to December 2018. Black: all zones, grey: rural zone, yellow: suburban zone and blue: urban zone.

From January 2017 to December 2018, *Ae. aegypti* and *Ae. albopictus* were detected in 693 water containers in Vassouras. Two containers were excluded from the analysis due to lack of date and address information. Other species besides *Ae. aegypti* and *Ae. albopictus* were detected in 53 of those containers (7.65%). The House (HI) and Breteau indexes (BI) were always below 1% for both vectors (Figure 4), which was classified as ‘satisfactory’ according to the criteria adopted by the Brazil Ministry of Health. In general, the BI had equal or similar values to the HI (Figure 4): in 2017, health agents found an average of 1.06 and 1.07 larval habitats per positive premise for *Ae. aegypti* and *Ae. albopictus*, respectively. In 2018, these averages were 1.10 and 1.11, respectively. These values indicate that only one positive water reservoir for immature mosquitoes was found in most of the surveyed houses.

**Figure 5:**
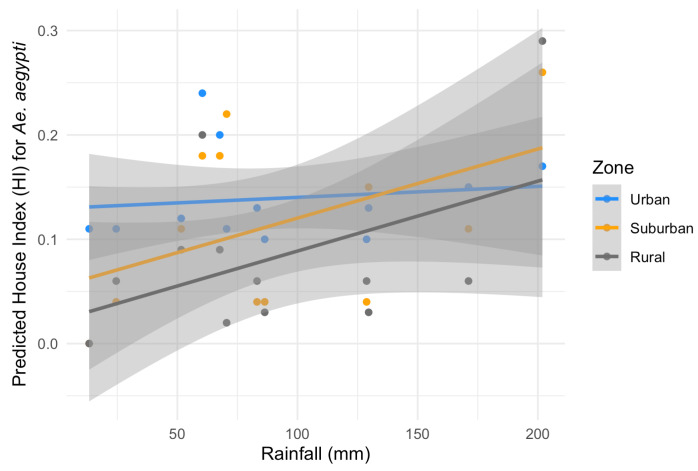
Predicted effects of zone and rainfall on the House index (HI) for *Aedes aegypti* with their 95% confidence intervals. Grey: rural zone, yellow: suburban zone and blue: urban zone.

**Figure 6:**
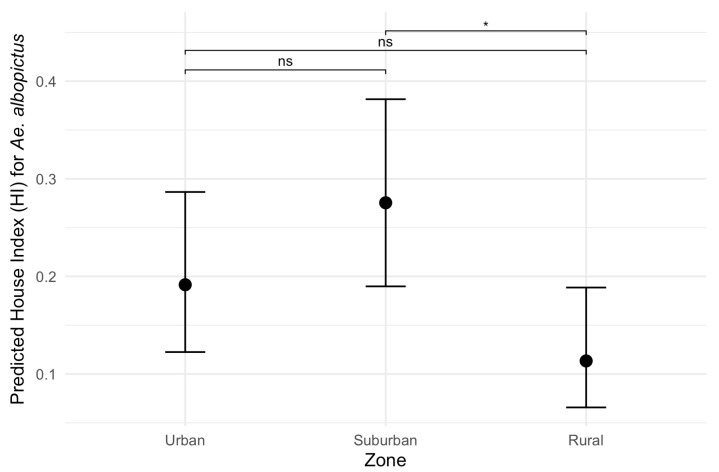
Predicted effects of zone on the House index (HI) for *Aedes albopictus*. Black dots represent estimated marginal means (EMMs) of House Index (HI) values along with their 95% confidence intervals. Pairwise comparisons: ns: non-significant; *: *p*-value < 0.05.

Considering the HI for *Ae. aegypti*, the best-fitting model included temperature, rainfall, zone and a significant interaction between zone and rainfall (ANOVA: zone*rainfall chi-square = 6.33; df = 2; *p*-value = 0.04). The interaction term indicates that the effect of rainfall on the HI varies across zones. Specifically, rainfall was positively associated with the HI in the suburban and rural zones (interaction terms: *p* = 0.02 and *p* = 0.04, respectively, Table 2) in contrast to the urban area (the reference level, Figure 5). This context-dependent relationship suggests that increases in rainfall lead to higher HI values primarily in suburban and rural areas (Figure 5). Additionally, pairwise comparisons of EMMs calculated using a mean rainfall value showed that the HI was significantly higher in the urban zone compared to the rural zone (*t* = 3.37, df = 28, *p* = 0.006) (Table 2, Figure 5). Regarding the HI of *Ae. albopictus*, the final model had only zone as an independent variable, which had a significant association with the HI (ANOVA: zone chi-square = 8.96; df = 2; *p*-value = 0.01), with significantly higher infestation in the suburban zone in comparison to the rural zone (EMM pairwise comparison: df = 32; *t* = 2.99; *p*-value = 0.01) (Table 3, Figure 6). Model selection details, ANOVAs and EMM pairwise comparisons can be found in Table S1. Finally, the comparison between mosquito species revealed that the HI was higher for *Ae. albopictus* than for *Ae. aegypti* in the suburban (estimated difference = 0.14; 95% CI: 0.08–0.21) and rural zones (estimated difference = 0.08; 95% CI: 0.02–0.17), while no difference was detected in the urban area (estimated difference = 0.02; 95% CI: −0.03–0.09).

### 3.3. Containers Types Used by Aedes aegypti and Ae. albopictus as Larval Habitats

From the 691 water containers considered for infestation analyses, 671 were classified into types A to E. In total, 273 and 398 water containers were found holding *Ae. aegypti* and *Ae. albopictus* immatures, respectively (Table 4). Analyzing containers per type, the number of larval habitats of type A was higher in the urban zone (the reference level) in comparison to the suburban area (GLM for type A—ANOVA: zone chi-square = 11.10, df = 2, *p*-value < 0.01). There was a significant positive association between the frequency of C and D larval habitats of *Ae. aegypti* and the mean temperature (GLMM for type C—ANOVA: temperature chi-square = 3.99, df = 1, *p*-value = 0.04; GLMM for type D—ANOVA: temperature chi-square = 4.91, df = 1, *p*-value = 0.03), while the frequency of A and B types was not related to the tested climatic variables (Table 5). Model selection details for *Ae. aegypti* data and ANOVA results can be found in Table S2.

Regarding *Ae. albopictus*, larval habitats of type A were also less abundant in the suburban zone in comparison to the urban area (GLM for type A—ANOVA: zone chi-square = 7.77, df = 2, *p*-value = 0.02). The same could be seen for B containers, which were also less abundant in the suburban in comparison to the urban zone (GLM for type B—ANOVA: zone chi-square = 12.1, df = 2, *p*-value < 0.01). Larval habitats of type B containers also had a positive association with the mean temperature (GLM for type B—ANOVA: temperature chi-square = 7.44, df = 2, *p*-value < 0.01). Finally, the number of type C containers had a positive association with the mean accumulated rainfall (GLMM for type C—ANOVA: rainfall chi-square = 9.47, df = 1, *p*-value < 0.01), while the frequency of the D type containers was not related to the tested climatic variables (GLMM for type D—ANOVA: rainfall chi-square = 3.97, df = 1, *p*-value = 0.05) (Table 6). Model selection details for *Ae. albopictus* data and ANOVA results can be found in Table S3.

**Figure 7:**
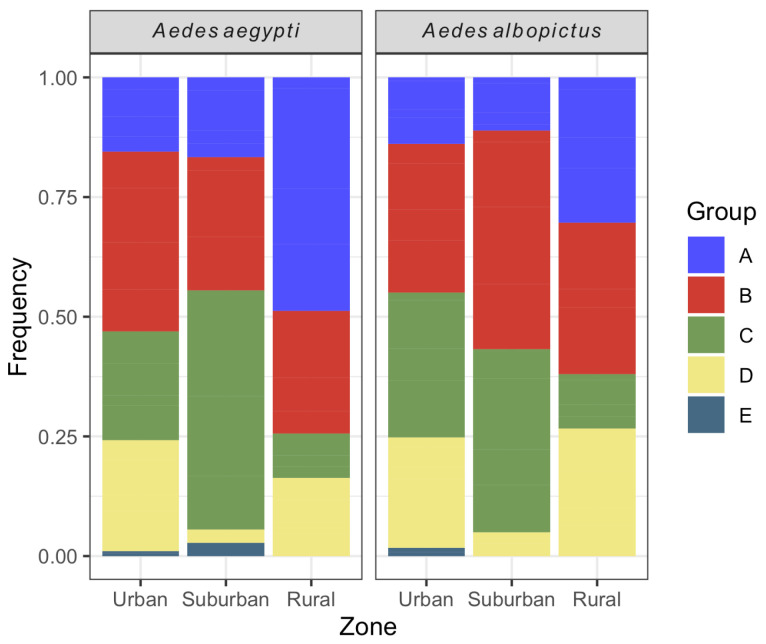
Relative frequency of *Aedes aegypti* and *Aedes albopictus* larval habitats per urbanization zone in Vassouras from January 2017 to December 2018.

The majority of *Ae. aegypti* larval habitats were classified into type B (34.4%) (mobile reservoirs) followed by type C (24.2%, fixed reservoirs). A similar profile was seen for *Ae. albopictus*, with 34.2 and 28.1% of containers classified into types B and C, respectively. The less frequent reservoirs for both species were those from type E (natural reservoirs). Both *Ae. aegypti* and *Ae. albopictus* exhibited significant differences in the relative frequency of water container types between urbanization zones (*Ae. aegypti:* chi-square = 40.0, df = 8, *p*-value < 0.001; *Ae. albopictus*: chi-square = 37.1, df = 8, *p*-value < 0.001). For *Ae. aegypti*, the proportion of B containers was higher in the urban area, while C and A containers were the majority in the suburban and rural zones, respectively. Regarding *Ae. albopictus*, the relative frequencies of B and C containers were the highest in the urban and suburban areas, while A and B containers were the majority in the rural zone. For both species, the relative abundance of D containers was lower in the suburban zone in comparison with the other areas. Natural larval habitats (E) were not found in the rural zone for both species (Figure 7).

### 3.4. Mapping Aedes aegypti and Ae. albopictus Larval Habitats

**Figure 8:**
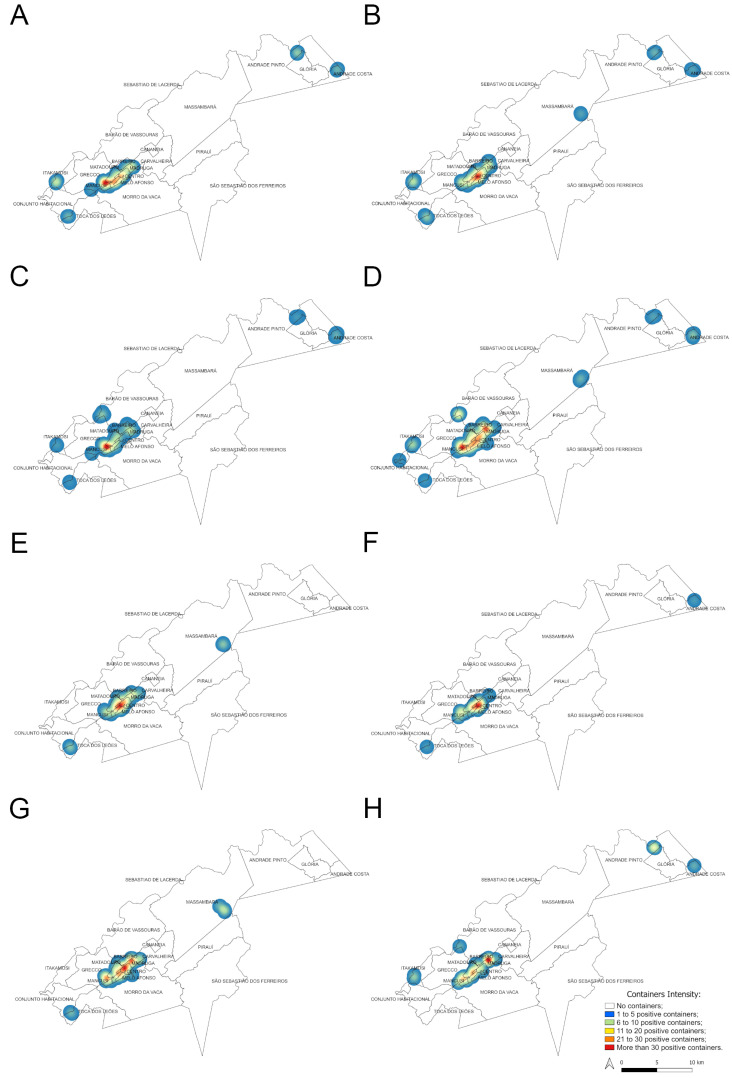
Heat (Kernel) maps showing the density of positive containers found during rainy season for *Ae. aegypti* ((**A**) for 2017 and (**B**) for 2018) and *Ae. albopictus* ((**C**) for 2017 and (**D**) for 2018) and during dry seasons for *Ae. aegypti* ((**E**) for 2017 and (**F**) for 2018) and *Ae. albopictus* ((**G**) for 2017 and (**H**) for 2018).

The Kernel maps (Figure 8) show a heterogeneous spatial distribution of positive water containers for *Ae. aegypti* and *Ae. albopictus* in the municipality of Vassouras. The highest concentration of positive containers for *Ae. aegypti* occurred in urban and suburban areas, especially in the neighborhoods of Santa Amalia, Centro and Madruga, irrespective of study years and seasons (Figure 2 and Figure 8A,B,E, F), where more than 30 *Ae. aegypti* larval habitats were found per season. This region has the highest concentration of houses. Other surrounding neighborhoods in the urban and suburban zones also exhibited positive containers overtime for this mosquito species but in a lower concentration (Figure 8A,B,E,F). During the rainy season, four larval habitat clusters were detected in the rural zone, which were constant between 2017 and 2018 (Figure 8A,B), except for one additional cluster in Massarambá that only occurred in 2018 (Figure 8B). On the other hand, only two clusters of positive containers for *Ae. aegypti* were detected during dry seasons, one of them was constant between years (in the Toca dos Leões neighborhood) while the other was variable (Figure 8E,F). All the clusters in the rural zone coincided with small house agglomerations and never exhibited more than 20 larval habitats (Figure 8A,B,E,F).

Considering *Ae. albopictus*, the highest concentration of positive containers (>30) was also in urban and suburban neighborhoods for both years and seasons (Figure 8C,D,G,H) but it was usually more widespread in space than observed for *Ae. aegypti*, comprising other suburban neighborhoods, such as Grecco (except for the rainy season of 2017, Figure 8C). In the rural area, clusters with a lower concentration of larval habitats (up to 20 positive containers) were seen: five to seven during the rainy season, most of them constant between 2017 and 2018 (Figure 8C,D). On the other hand, only two clusters of *Ae. albopictus* were detected during dry seasons which were different between 2017 and 2018 (Figure 8G,H). As seen for *Ae. aegypti*, clusters in the rural zone coincided with small house agglomerations.

## 4. Discussion

The present study sought to investigate and contrast the infestation of *Ae. aegypti* and *Ae. albopictus* at different levels of urbanization in Vassouras, RJ, as well to address which water reservoirs had been used by each species as larval habitats. The House Index (HI) was always low (<1%) for both mosquito species [29] and was positively associated with rainfall for *Ae*. *aegypti* in suburban and rural zones. Infestation (HI) was higher for *Ae. albopictus* than for *Ae. aegypti* in suburban and rural areas, with more B and C reservoirs as the most common types of habitat for both species. For both vectors, the relative distribution of containers into different types varied across urbanization zones. Finally, the spatial distribution of larval habitats was similar between species, as well as often constant over time.

In 2017 and 2018, the HI and BI for *Ae. aegypti* and *Ae. albopictus* were always low (<1%), classified as ‘satisfactory’ according to the criteria adopted by the Brazil Ministry of Health. Although a HI of <1% has traditionally been considered indicative of low risk for arbovirus epidemics [38,39], it may not indicate the absence of arbovirus transmission, since DENV and CHIKV human infections were detected during the study. Arbovirus transmission may still occur in areas where highly productive larval habitats persist, or where even low vector densities coincide with continuous viral introduction, high vector capacity, favorable climatic conditions and/or susceptible host populations. Thus, larval-based indices, while useful for operational purposes, may have limited predictive value for real-time epidemiological risk and should be interpreted with caution [40].

The HI of *Ae. aegypti* was positively associated with rainfall, especially in the suburban and rural zones. Environmental factors, such as precipitation, have also been related to the higher proliferation and seasonal population fluctuation of this species under distinct climates in the Americas, as seen in subtropical Argentina [41] and tropical Southeast Brazil [42] and Mexico [43]. Precipitation was also a predominant climatic variable associated with the increase in dengue incidence in Brazil [44,45] probably due to the greater availability of water reservoirs for mosquito development during the rainy periods. On the other hand, we did not detect significant correlations between the HI and temperature, although the number of larval habitats from types C and D were positively associated with this climatic variable. As seen before in other investigations from South America, the prominent increase in mosquito infestation in warmer temperatures usually occurs in subtropical and temperate regions such as in Southern Brazil [46] and Argentina [47,48,49], where the cold winter may limit mosquito survival and development. Vassouras has a favorable temperature for the proliferation of *Ae. aegypti* and *Ae. albopictus* during the entire year (ranging from >15 to 30 °C on average) [50,51] but some habitat types may be more exposed to temperature fluctuations, which can affect larval survival [52].

Two previous studies investigated the presence of *Ae. aegypti* and *Ae. albopictus* in the city. Using tires as larval traps or ovitraps in urban and suburban neighborhoods, both species were also detected with higher abundance during the rainy season, from December to March [53,54]. In this sense, local climatic data can be valuable indicators for anticipating increases in mosquito populations, supporting early warning systems and spatial risk assessments for arbovirus outbreaks. This information is crucial for guiding more effective and targeted vector control interventions [42]. Furthermore, these findings underscore the importance of strengthening entomological surveillance and control measures during the rainy season, including public education campaigns to raise awareness about the elimination or reduction of potential larval habitats for *Aedes* mosquitoes [55].

Often, the BI was equal or similar to the HI, i.e., health agents usually found one larval habitat per house. According to Ribeiro et al., 2021 [56], this situation suggests that the inspection was interrupted after the identification of the first larval habitat with *Aedes* immatures. The authors suggest that this may be due to a lack of knowledge of health agents regarding the relevance of the BI and point to the need for permanent investment in training for those carrying out larval surveys. Since vector control, information and communication measures for the prevention of arboviruses are guided by records of the types of dominant larval habitats in each territory, biases in the results can lead to mistaken measures on the part of public health managers [56].

Although we did not evaluate the abundance of *Aedes* immatures, as five specimens were sampled per larval habitat, a greater number of containers were found harboring *Ae. albopictus* compared to *Ae. aegypti* from 2017 to 2018, with a significantly higher HI for *Ae. albopictus* in suburban and rural zones. This fact is possibly related to the ecology of *Ae. albopictus*, which inhabits more vegetated environments with a colder climate when compared to *Ae. aegypti* [57,58]. The municipality of Vassouras has extensive rural and suburban areas, interspersed with regions of intense vegetation cover. Furthermore, it has milder temperatures than other cities in Rio de Janeiro, especially those in the metropolitan region (with large urban concentration), which are normally more infested by *Ae. aegypti* [59].

In accordance with this, although the two species coexist during most larval surveys in all areas, the HI of *Ae. aegypti* was predominantly higher in the urban zone, while the *Ae. albopictus* HI was usually higher in the suburban area. This observation is consistent with previous studies showing that *Ae. aegypti* is a highly anthropophilic mosquito, which is usually found close to human dwellings, whereas *Ae. albopictus* prefers less urbanized areas [59,60,61,62,63,64,65]. Despite this, the absolute number of larval habitats was higher for both species in the urban area, a region that has the highest concentration of people and a number of premises that is up to three times greater than the others. This scenario may result in more suitable conditions for mosquito breeding and arbovirus transmission in urbanized regions, but, as seen before by Overgaard et al. [63], rural infestation is also substantial and cannot be ignored by vector control agencies. In a similar study, Braks et al. [16] concluded that in Brazil, rural areas may not be avoided by *Ae. aegypti*, as the availability indoors of human hosts and domestic containers is similar to urban areas.

Infestation levels (HI) for *Ae. aegypti* fluctuated more evidently according to the rainfall in suburban and rural zones when compared to the urban area. This could be due to the more frequent use of containers inside human houses in the urban area as larval habitats, while mosquitoes would often oviposit in containers around homes in the suburban and rural zones [16,59,66,67]. So, in more urbanized neighborhoods, *Ae. aegypti* would frequently use domestic larval habitats that are less dependent on rainwater, such as plant vases, thaw containers from refrigerators and animal drinking fountains (type B), and rainfall would not be a primary factor in the proliferation of this mosquito. On the other hand, in suburban and rural places it would often oviposit in water reservoirs located in open areas, which are more dependent on rainwater [61,68], such as type C (gutters, disused toilets, non-treated pools—abundant in suburban neighborhoods) or A reservoirs (storage containers for domestic use which frequently collect rain water—abundant in rural areas). This scenario would contribute to infestation levels fluctuating more sharply according to the pluviosity in specific zones [69,70]. The main container types available are believed to explain the differences between urban and rural mosquito production [63].

Considering all the city zones together, most larval habitats of *Ae. aegypti* and *Ae. albopictus* were peridomestic reservoirs classified into types B (mobile reservoirs such as vases, bottles and religious objects), followed by C (fixed containers, e.g., tanks, disused toilets and non-treated pools) in accordance with Wilson-Bahun et al. [65] but contrasting previous reports that water reservoirs for domestic use (type A) are the most prevalent containers holding *Ae. aegypti* larvae [71,72]. Nonetheless, these findings must be interpreted with caution, as we cannot determine if they represent the most productive container type, since our sampling strategy only enabled us to identify the positive water reservoirs for each species but did not measure their abundance, as five larvae were identified from each larval habitat. Our study was developed within the routine surveillance activities conducted by the local health agents, and this sample size aimed to ensure the logistical feasibility of large-scale field surveys conducted every two months in the city.

Another limitation of the study is that our data does not allow us to say how many of the domestic containers had the two species coexisting, as information regarding larvae collection from each positive container was registered into species-specific datasets, following the standard routine used by the municipal surveillance program. Even so, the similarity in the larval habitat profile between *Ae. aegypti* and *Ae. albopictus* is in accordance with the knowledge that these vectors coexist in the anthropic environment, sharing larval habitats [73,74,75]. In the same way, other culicids species common in domestic water reservoirs, such as *Culex quinquefasciatus*, were registered as “other” during larval surveys, following the *Aedes* surveillance protocol adopted in Brazil [29]. This is because *Ae. aegypti* and *Ae. albopictus* are the known vectors of arboviruses such as dengue, Zika and chikungunya in Brazil. Nevertheless, given the relevance of ecological interactions and resource competition between culicids in larval habitats to vector population dynamics [76], we recommend that surveillance programs should include protocols to record species coexistence at the container level, as well as other mosquito taxa besides *Aedes*.

Natural recipients (type E) were the least found for both species (~1% of larval habitats). This was unexpected, since *Ae. albopictus* is believed to oviposit in both artificial and natural water reservoirs, such as tree holes and leaf axils [17,68]. Perhaps this observation results from the fact that collections were made in premises but not in forest parks, squares and botanical gardens, where *Ae. albopictus* have been occasionally collected in natural water reservoirs in Brazil [77,78,79]. In any case, our data indicate that in Vassouras, (i) natural containers are not common larval habitats for both vector species inside and around human households and (ii) *Ae. albopictus* successfully colonizes urbanized, suburban and rural areas, where it breeds mostly in artificial water containers. As seen elsewhere, the establishment of this mosquito in anthropic environments [80,81] could suggest the beginning of its domiciliation, as occurred before with *Ae. aegypti* [74].

The distribution of water containers into the five types differed between urbanization zones for both vectors. For example, there was an increase in the proportion of type A recipients (represented by large water storage reservoirs for domestic use) in the rural zone. In addition, similar numbers of type A larval habitats were found in rural areas compared to urban areas (considering the two species), despite the first area having half the number of premises compared to the second. Intermittent water supply in rural neighborhoods (which does not occur in urban and suburban areas in Vassouras) means that the population needs to store it for domestic consumption in barrels or needs to keep water tanks uncovered to collect rainwater. Thus, these containers often become perennial larval habitats for *Ae. aegypti* and *Ae. albopictus* [66,82]. Perhaps because of their constant availability regardless of rainfall or temperature fluctuations, the amount of this type of habitat was the only one that showed no significant association with the tested climatic variables for either species.

Another fact that draws attention is the lower relative abundance of type D larval habitats (discarded tires) in the suburban area in relation to the others. In Vassouras, used tires are usually used in rural areas to build barriers (retaining walls) and may become oviposition sites for *Aedes* mosquitoes. The disposal of this material in neighborhoods further away from the city center is also problematic. In the urban area there are some tire shops and warehouses where tires are stored and can become *Aedes* larval habitats. The urban landscape and human behavior might have implications in vector ecology, including water reservoirs used by domestic mosquitoes. Thus, vector control activities and education campaigns for arbovirus prevention must be neighborhood/zone-specific [23].

In general, the spatial distribution of larval habitats was similar between species, seasons and over the two years of study, i.e., we did not detect any habitat segregation between species, neither in space nor time. The area with the highest concentration of larval habitats occurred overtime in the same urban and suburban neighborhoods, such as Santa Amália, Centro and Madruga, where there is the highest agglomeration of houses, reinforcing the adaptation of both species to the anthropic environment [16]. Moreover, since a greater proportion of positive containers supposes a greater abundance of mosquitoes, this area would be at higher risk for arbovirus transmission, allowing for data-driven decision-making by public health authorities, such as targeted entomological surveillance and vector control activities [83].

In this study, the presence of *Ae. aegypti* and *Ae. albopictus* was confirmed in all city zones, with the former predominating in urbanized areas whereas the latter was more frequent in suburban zones, making it clear that these species can coexist within urban-gradients, as pointed out before by [16,58]. In this context, unplanned urban development, often lacking adequate infrastructure for waste management and water supply, facilitates the proliferation of these vector species. Overall, both species were associated with similar types of artificial containers, but limitations in the methodology prevented the direct assessment of species co-occurrence in individual containers. Detecting vector spatial distribution and understanding their breeding ecology, including their oviposition habits in different urban landscapes, enables public health managers to apply vector control measures and education strategies that are more specific and effective to mitigate the proliferation of these insects. Moreover, key neighborhoods with persistent *Aedes*-positive containers were identified, providing valuable information for targeting control efforts and improving local vector surveillance strategies. This is still the best way to prevent diseases caused by arboviruses transmitted by *Ae. aegypti* and *Ae. albopictus*.

## Figures and Tables

**Table 1:** Common characteristics of neighborhoods according to urbanization zones of Vassouras Municipality, Rio de Janeiro, Brazil.

Zone	Piped Water Supply and Garbage Collection	Paved Streets	Peridomestic Area of Premises
Urban	Regular	Yes	Yes, decorated backyards
Suburban	Regular	Yes	Yes, intermediate size between urban and rural areas
Rural	Irregular	No	Yes, pastures and cultivation areas

**Table 2:** Results of the final beta regression model testing the effects of temperature, rainfall and city zone on the House Index (HI) for *Aedes aegypti*.

Dependent Variable	Independent Variables	Estimate	Std. Error	z-Value	*p*-Value
House Index for *Ae. aegypti*	Intercept	−3.72	1.63	−2.29	0.02
	Temperature	0.11	0.09	1.23	0.21
	Rainfall	−0.003	0.004	−0.64	0.52
	**Zone (Suburban)**	**−1.52**	**0.51**	**−2.97**	**0.002**
	**Zone (Rural)**	**−1.85**	**0.56**	**−3.29**	**0.001**
	Precipitation*Zone (Suburban)	**0.01**	**0.004**	**2.24**	**0.02**
	Precipitation*Zone (Rural)	**0.01**	**0.005**	**2.08**	**0.04**

The reference level for zone is ‘urban’. Temperature and rainfall were averaged bimonthly. Number of larval surveys = 12. Number of larval habitats used in HI calculations = 283. * Interaction term between independent variables. Variables with significant effect according to ANOVA are shown in bold.

**Table 3:** Results of the final beta regression model testing the effects of temperature, rainfall and city zone on the House Index (HI) for *Aedes albopictus*.

Dependent Variable	Independent Variables	Estimate	Std. Error	z-Value	*p*-Value
House Index for *Ae. albopictus*	Intercept	−1.44	0.26	−5.55	<0.01
	Zone (Suburban)	0.47	0.34	1.38	0.17
	Zone (Rural)	−0.61	0.37	−1.66	0.09

The reference level for zone is ‘urban’. Number of larval surveys = 12. Number of larval habitats used in HI calculations = 408.

**Table 4:** Water containers holding *Aedes aegypti* and *Aedes albopictus* immatures per type and zone.

Species	Zone	A (%)	B (%)	C (%)	D (%)	E (%)	TOTAL (%)
*Aedes aegypti*	Urban	30	73	44	45	2	194 (71.1)
	Suburban	6	10	18	1	1	36 (13.2)
	Rural	21	11	4	7	0	43 (15.8)
	Total (%)	57 (20.9)	94 (34.4)	66 (24.2)	53 (19.4)	3 (1.1)	273 (100)
*Aedes albopictus*	Urban	33	74	72	55	4	237 (59.5)
	Suburban	9	37	31	4	0	82 (20.6)
	Rural	24	25	9	21	0	79 (19.8)
	Total (%)	66 (16.6)	136 (34.2)	112 (28.1)	80 (20.1)	4 (1.0)	398 (100)

**Table 5:** Results of the final generalized linear mixed model (GLMM, negative binomial) testing the effects of temperature, rainfall and species on the amount of each type (A, B, C and D) of positive containers for *Aedes aegypti* immatures.

Container Type	Effects	Independent Variables	Estimate	Std. Error	z-Value	*p*-Value
A (water storage) N = 57	Fixed	Intercept	0.92	0.22	4.10	<0.01
		**Zone (Suburban)**	**−1.61**	**0.48**	**−3.33**	**<0.01**
		Zone (Rural)	−0.36	0.34	−1.05	0.29
B (mobile reservoirs) N = 94	Fixed	Intercept	−1.65	1.34	−1.20	0.22
		Temperature	0.10	0.06	1.80	0.07
	Random	Zone (variance)	0.84	-	-	-
C (fixed reservoirs) N = 66	Fixed	Intercept	−2.23	1.38	−1.61	0.10
		**Temperature**	**0.12**	**0.06**	**2.00**	**0.04**
	Random	Zone (variance)	0.85	-	-	-
D (removable reservoirs) N = 53	Fixed	Intercept	−3.51	1.66	−2.11	0.03
		**Temperature**	**0.14**	**0.06**	**2.22**	**0.03**
	Random	Zone (variance)	2.17	-	-	-

The reference level for zone is ‘urban’. ‘N’ is the number of larval habitats from each type. Temperature and rainfall were averaged bimonthly. Number of larval surveys = 12. Variables with significant effect according to ANOVA are shown in bold.

**Table 6:** Results of the final generalized linear mixed model (GLMM, negative binomial) testing the effects of temperature, rainfall and species on the amount of each type (A, B, C and D) of positive containers for *Aedes albopictus* immatures.

Container Type	Effects	Independent Variables	Estimate	Std. Error	z-Value	*p*-Value
A (water storage) N = 66	Fixed	Intercept	−0.50	2.07	−0.24	0.81
		Temperature	0.046	0.11	0.41	0.68
		Rainfall	0.005	0.004	1.12	0.26
		**Zone (Suburban)**	**−1.27**	**0.46**	**−2.75**	**<0.01**
		Zone (Rural)	−0.22	0.38	−0.58	0.56
B (mobile reservoirs) N = 136	Fixed	Intercept	−2.81	1.63	−1.73	0.08
		**Temperature**	**0.24**	**0.09**	**2.73**	**<0.01**
		Rainfall	−0.004	0.003	−1.28	0.20
		**Zone (Suburban)**	**−0.71**	**0.31**	**−2.29**	**0.02**
		**Zone (Rural)**	**−1.10**	**0.33**	**−3.32**	**<0.01**
C (fixed reservoirs) N = 112	Fixed	Intercept	0.16	0.52	0.30	0.76
		**Rainfall**	**0.006**	**0.002**	**3.08**	**<0.01**
	Random	Zone (variance)	0.60	-	-	-
D (removable reservoirs) N = 80	Fixed	Intercept	−0.35	0.71	−0.49	0.62
		Rainfall	0.007	0.003	1.99	0.046
	Random	Zone (variance)	0.92	-	-	-

The reference level for zone is ‘urban’. ‘N’ is the number of larval habitats from each type. Temperature and rainfall were averaged bimonthly. Number of larval surveys = 12. Variables with significant effect according to ANOVA are shown in bold.

## Data Availability

The raw data supporting the conclusions of this article will be made available by the authors on request.

## Supplementary Materials

The following supporting information can be downloaded at: https://www.mdpi.com/article/10.3390/insects16080869/s1, Table S1: Model selection for beta analysis for House Index (data) (A), ANOVA test for final beta regression models (B), Variance Inflation Factors (VIFs) (C) and Estimated Marginal Means (EMMs) pairwise comparison of HI between zones (D); Table S2: Model selection for Generalized Linear (Mixed) Models (GLMMs) for the frequency of each type of *Aedes aegypti* larval habitat (A), ANOVA test for final models (B) and Variance Inflation Factors (VIFs) (C); Table S3: Model selection for Generalized Linear (Mixed) Models (GLMMs) for the frequency of each type of *Aedes albopictus* larval habitat (A), ANOVA test for final models (B) and Variance Inflation Factors (VIFs) (C).

## Abbreviations

DENV	Dengue virus
CHIKV	Chikungunya virus
ZIKV	Zika virus
MHS	Municipal Health Secretariat
HI	House Index
BI	Breteau Index
GLM	Generalized Linear Model
GLMM	Generalized Linear Mixed Model
PNCD	Brazilian Dengue Control Program
df	Degrees of freedom
