# Supplementary material for: Infestation and Larval Habitat Ecology of Aedes aegypti and Aedes albopictus in an Urban Gradient in Vassouras, Rio de Janeiro, Brazil
Source: Insects. 2025 Aug 21;16(8):869. doi: 10.3390/insects16080869 (PMC12386281; doi:10.3390/insects16080869)
Supplement: Supplementary file 1 [file insects-16-00869-s001.zip › insects-3598344-supplementary.pdf]

Table S1: Model selection for beta analysis for House Index (data) (A), ANOVA test for final beta regression models (B), Variance Inflation Factors (VIFs) (C) and Estimated Marginal Means (EMM) pairwise comparison of HI between zones (D).

A) Beta regression model selection:

Beta Regression Model (for *Aedes aegypti*)	df	AIC	
HI_aeg ~ 1 [a]	2	-84.14	
HI_aeg ~ 1 + (1 ∣ Zone) [a]	3	-83.71	
HI_aeg ~ Temperature	3	-92.46	
HI_aeg ~ Rainfall	3	-93.01	
HI_aeg ~ Zone	4	-87.81	
HI_aeg ~ Temperature + Rainfall + Zone	6	-94.94	
HI_aeg ~ Temperature + Rainfall + Zone + Zone*Temperature	8	-94.68	
**HI_aeg ~ Temperature + Rainfall + Zone + Zone*Rainfall**	**8**	**-96.86**	Final model
HI_aeg ~ Temperature + Rainfall + Zone + Zone*Temperature + Zone*Rainfall	10	-93.85	

Beta Regression Model (for *Aedes albopictus*)	df	AIC	
HI_alb ~ 1 [a]	2	-44.23	
HI_alb ~ 1 + (1 ∣ Zone) [a]	3	-44.29	
HI_alb ~ Temperature	3	-43.3	
HI_alb ~ Rainfall	3	-43.77	
**HI_alb ~ Zone**	**4**	**-48.63**	Final model
HI_alb ~ Temperature + Rainfall + Zone	6	-47.43	
HI_alb ~ Temperature + Rainfall + Zone + Zone*Temperature	8	-46.83	
HI_alb ~ Temperature + Rainfall + Zone + Zone*Rainfall	8	-44.96	
HI_alb ~ Temperature + Rainfall + Zone + Zone*Temperature + Zone*Rainfall	10	-43.92	

[a] Models with Zone as a random intercept and as a fixed effect were compared to support the assumption of independence between observations. A substantial improvement in model fit ($\Delta AIC > 2$) when including Zone as a random effect would suggest that observations are not independent across zones and that hierarchical structure should be accounted for in the analysis.

B) Beta regression ANOVA:

Analysis of Deviance Table (Type III Wald chi squared tests)

Beta Regression Model (for Aedes aegypti): HI_aeg ~ Temperature + Rainfall + Zone + Zone*Rainfall	Chisq	df	p-value
Temperature	1.5	1	0.22
Rainfall	0.4	1	0.52
**Zone**	**13.98**	**2**	**0.0009**
**Rainfall*Zone**	**6.33**	**2**	**0.04**

Analysis of Deviance Table (Type II Wald chi squared tests)

Beta Regression Model (for *Aedes albopictus*): HI_alb ~ Zone	Chisq	df	p-value
**Zone**	**8.96**	**2**	**0.011**

C) Variance Inflation Factors (VIFs):

Beta Regression Model (for Aedes aegypti): HI_aeg ~ Temperature + Rainfall + Zone + Zone*Rainfall	Df	GVIF^(1/(2*Df))
Temperature	1	1.54
Rainfall	1	2.09
Zone	2	1.94
Zone*Rainfall	2	2.16

Table S2: Model selection for Generalized Linear (Mixed) Models (GLMM) for the frequency each type of *Aedes aegypti* larval habitat (A), ANOVA test for final models (B) and Variance Inflation Factors (VIFs) (C).

A) GLM/GLMM model selection for *Ae. aegypti*:

**Containers Type A**	df	AIC	
Freq_A ~ 1 [a]	2	127.30	
Freq_A ~ 1 + (1 ∣ Zone) [a]	3	125.57	
Freq_A ~ Temperature	3	128.85	
Freq_A ~ Rainfall	3	128.72	
**Freq_A ~ Zone**	**4**	**119.57**	Final model
Freq_A ~ Temperature + Rainfall + Zone	6	122.52	
Freq_A ~ Temperature + Rainfall + Zone + Temperature*Zone	8	124.56	
Freq_A ~ Temperature + Rainfall + Zone + Rainfall*Zone	8	125.94	
Freq_A ~ Temperature + Rainfall + Zone + Temperature*Zone + Rainfall*Zone	10	128.37	

**Containers Type B**	df	AIC	
Freq_B ~ 1 [a]	2	156.71	
Freq_B ~ 1 + (1 ∣ Zone) [a]	3	139.36	
**Freq_B ~ Temperature + (1 ∣ Zone)**	**4**	**138.16**	Final model
Freq_B ~ Rainfall + (1 ∣ Zone)	4	141.20	
Freq_B ~ Temperature + Rainfall + (1 ∣ Zone)	5	NA	

**Containers Type C**	df	AIC
Freq_C ~ 1 [a]	2	136.32
Freq_C ~ 1 + (1 ∣ Zone) [a]	3	123.50

**Freq_C ~ Temperature + (1 ǀ Zone)**	**4**	**121.33**	Final model
Freq_C ~ Rainfall + (1 ǀ Zone)	4	123.43	
Freq_C ~ Temperature + Rainfall + (1 ǀ Zone)	5	123.31	

**Containers Type D**	df	AIC	
Freq_D ~ 1 [a]	2	121.17	
Freq_D ~ 1 + (1 ǀ Zone) [a]	3	98.99	
**Freq_D ~ Temperature + (1 ǀ Zone)**	**3**	**95.68**	Final model
Freq_D ~ Rainfall + (1 ǀ Zone)	3	97.07	
Freq_D ~ Temperature + Rainfall + (1 ǀ Zone)	6	NA	

[a] Models with Zone as a random intercept and as a fixed effect were compared to support the assumption of independence between observations. A substantial improvement in model fit (ΔAIC > 2) when including Zone as a random effect would suggest that observations are not independent across zones, thus, model selection proceeded with a hierarchical structure.

NA: Convergence issues were observed in models due to the proportion of zero counts.

B) GLM/GLMM ANOVA:

Analysis of Deviance Table (Type II Wald chi squared tests)			
Type A: Freq_A ~ Zone	Chisq	Df	p-value
**Zone**	**11.1**	**2**	**0.004**

Analysis of Deviance Table (Type II Wald chi squared tests)			
Type B: Freq_B ~ Temperature + (1 ǀ Zone)	Chisq	Df	p-value
Temperature	3.24	1	0.07

Analysis of Deviance Table (Type II Wald chi squared tests)			
Type C: Freq_C ~ Temperature + (1 ǀ Zone)	Chisq	Df	p-value
**Temperature**	**3.99**	**1**	**0.04**

Analysis of Deviance Table (Type II Wald chi squared tests)			
Type D: Freq_D ~ Temperature + (1 ǀ Zone)	Chisq	Df	p-value
**Temperature**	**4.91**	**1**	**0.03**

C) GLMM Variance Inflation Factors (VIFs):

All larval habitat types:	VIF
Temperature	2.36
Rainfall	2.36

Table S3: Model selection for Generalized Linear (Mixed) Models (GLMM) for the frequency each type of *Aedes albopictus* larval habitat (A), ANOVA test for final models (B) and Variance Inflation Factors (VIFs) (C).

A) GLM/GLMM model selection for *Ae. albopictus*:

**Containers Type A**	df	AIC	
Freq_A ~ 1 [a]	2	136.41	
Freq_A ~ 1 + (1 ∣ Zone) [a]	3	137.56	
Freq_A ~ Temperature	3	135.53	
Freq_A ~ Rainfall	3	135.08	
Freq_A ~ Zone	4	133.62	
**Freq_A ~ Temperature + Rainfall + Zone**	**6**	**133.26**	Final model
Freq_A ~ Temperature + Rainfall + Zone + Temperature*Zone	8	134.55	
Freq_A ~ Temperature + Rainfall + Zone + Rainfall*Zone	8	136.53	
Freq_A ~ Temperature + Rainfall + Zone + Temperature*Zone + Rainfall*Zone	10	135.64	

**Containers Type B**	df	AIC	
Freq_B ~ 1 [a]	2	180.06	
Freq_B ~ 1 + (1 ∣ Zone) [a]	3	180.20	
Freq_B ~ Temperature	3	177.46	
Freq_B ~ Rainfall	3	181.17	
Freq_B ~ Zone	4	176.09	
**Freq_B ~ Temperature + Rainfall + Zone**	**6**	**172.40**	Final model
Freq_B ~ Temperature + Rainfall + Zone + Temperature*Zone	8	176.02	
Freq_B ~ Temperature + Rainfall + Zone + Rainfall*Zone	8	176.33	
Freq_B ~ Temperature + Rainfall + Zone + Temperature*Zone + Rainfall*Zone	10	179.42	

**Containers Type C**	df	AIC	
Freq_C ~ 1 [a]	2	167.99	
Freq_C ~ 1 + (1 ∣ Zone) [a]	3	157.26	
Freq_C ~ Temperature + (1 ∣ Zone)	4	152.49	
**Freq_C ~ Rainfall + (1 ∣ Zone)**	**4**	**151.53**	Final model
Freq_C ~ Temperature + Rainfall + (1 ∣ Zone)	5	152.92	

**Containers Type D**	df	AIC
Freq_D ~ 1 [a]	2	144.595
Freq_D ~ 1 + (1 ∣ Zone) [a]	3	139.22

Freq_D ~ Temperature + (1 ∣ Zone)	4	138.77	
**Freq_D ~ Rainfall + (1 ∣ Zone)**	**4**	**137.45**	Final model
Freq_D ~ Temperature + Rainfall + (1 ∣ Zone)	5	139.40	

[a] Models with Zone as a random intercept and as a fixed effect were also compared to support the assumption of independence between observations. A substantial improvement in model fit (ΔAIC > 2) when including Zone as a random effect would suggest that observations are not independent across zones, thus, model selection proceeded with a hierarchical structure.

NA: Convergence issues were observed in models due to the proportion of zero counts.

B) GLM/GLMM ANOVA:

Analysis of Deviance Table (Type II Wald chi squared tests)			
Type A: Freq_A ~ Temperature + Rainfall + Zone	Chisq	Df	p-value
Temperature	1.25	1	0.26
Rainfall	0.17	1	0.68
**Zone**	**7.77**	**2**	**0.02**

Analysis of Deviance Table (Type II Wald chi squared tests)			
Type B: Freq_B ~ Temperature + Rainfall + Zone	Chisq	Df	p-value
**Temperature**	**7.44**	**1**	**0.006**
Rainfall	1.63	1	0.2
**Zone**	**12.1**	**2**	**0.002**

Analysis of Deviance Table (Type II Wald chi squared tests)			
Type C: Freq_C ~ Rainfall + (1 ∣ Zone)	Chisq	Df	p-value
**Rainfall**	**9.47**	**1**	**0.002**

Analysis of Deviance Table (Type II Wald chi squared tests)			
Type D: Freq_D ~ Temperature + (1 ∣ Zone)	Chisq	Df	p-value
**Rainfall**	**3.97**	**1**	**0.05**

C) GLMM Variance Inflation Factors (VIFs):

All larval habitat types:	VIF
Temperature	2.36
Rainfall	2.36
